# Association Between β-thalassemia and Atrial Fibrillation: Insights from the United States National Inpatient Sample

**DOI:** 10.19102/icrm.2023.14095

**Published:** 2023-09-15

**Authors:** Wael Abdelmottaleb, Ahmed Maraey, Mustafa Ozbay, Hadeer Elsharnoby, Mahmoud Khalil, Andrea Natale, Mohamed Al Rawahi

**Affiliations:** ^1^Department of Medicine, Metropolitan Hospital Center, New York, NY, USA; ^2^Department of Internal Medicine, CHI St. Alexius Health, Bismarck, ND, USA; ^3^Department of Internal Medicine, Carle Foundation, Urbana, IL, USA; ^4^Department of Internal Medicine, Lincoln Hospital, Bronx, NY, USA; ^5^Texas Cardiac Arrhythmia Institute, Austin, TX, USA; ^6^Department of Medicine, Cardiology Unit, Sultan Qaboos University Hospital, Muscat, Oman

**Keywords:** Atrial fibrillation, national inpatient sample, thalassemia

## Abstract

Transfusion-dependent β-thalassemia (thalassemia major and thalassemia intermedia) (BT) requires repeated blood transfusions for survival due to ineffective erythropoiesis. Consequently, iron overload can predispose the patient to atrial fibrillation (AF) despite the improved prognosis achieved with transfusion and chelation therapy. We sought to study the impact of AF on BT patients through a large database analysis. The current study used data from the Agency for Healthcare Research and Quality’s Healthcare Cost and Utilization Project National Inpatient Sample collected from 2016–2019. A total of 17,150 admissions were included, of which 2100 (12.2%) admissions had a concomitant diagnosis of AF. Admissions with AF were older (mean age, 72.1 vs. 47.3 years; *P* < .001) and more likely to have congestive heart failure (CHF), hypertension, valvular heart disease, and renal disease. BT admission was associated with a higher AF prevalence than non-BT admission across all age groups. AF was not associated with an increased risk of in-hospital mortality (adjusted odds ratio [aOR], 1.36; 95% confidence interval [CI], 0.67–2.78; *P* = .398) or an increased length of stay (LOS) (aOR, 1.00; 95% CI, 0.78–1.29; *P* = .997) in the general cohort. In a subgroup analysis, AF was associated with increased in-hospital mortality in women (aOR, 2.73; 95% CI, 1.09–6.8; *P* = .031). Predictors of in-hospital mortality were increasing age, CHF, and liver disease, while predictors of prolonged LOS were diabetes mellitus, CHF, and increasing age. Further studies are warranted to develop strategies to improve the quality of care and outcome in this population.

## Background

β-thalassemia (BT) is a hereditary disease caused by absent or decreased β-globin chain synthesis during hemoglobin production. Depending on the degree of β-globin synthesis dysfunction, it is classified as thalassemia major, thalassemia intermedia, or thalassemia minor. Transfusion-dependent thalassemia (thalassemia major and intermedia) requires lifelong blood transfusions at regular intervals due to ineffective erythropoiesis and chronic anemia.

Before the introduction of chelation therapy, the most common cause of death in these patients receiving regular transfusions in the 1960s was congestive heart failure (CHF). After the initiation of chelation therapy with deferoxamine, mortality was postponed considerably, but mortality from cardiac iron overload continued to dominate the causes of death, accounting for about 70% of cases.^1^ Transfusion and chelation therapy have improved the prognosis and survival but led to new comorbidities such as atrial fibrillation (AF). Prior studies showed a higher prevalence of AF in patients with BT (2%–33%) compared to the general population (2%–4%). The major postulated mechanism is myocardial iron overload from repetitive transfusion, leading to cardiomyopathy, atrial dilatation, and remodeling. We sought to study the impact of AF on BT patients through a large database analysis.^2^

## Methods

The current study used data from the Agency for Healthcare Research and Quality’s Healthcare Cost and Utilization Project National Inpatient Sample collected from 2016–2019.^3^ BT admissions aged ≥18 years were included. Admissions with missing data were excluded from the analysis. BT admissions were identified using a relevant International Classification of Diseases, 10^th^ Edition, Clinical Modification (ICD-10-CM) code (D56.1). Admissions were stratified according to the presence or absence of AF. A series of multivariate logistic regressions were performed to evaluate the impact of AF on BT and to account for potential confounders. Continuous variables were compared using Student’s *t* test, and categorical variables were compared using the chi-squared test. A subgroup analysis according to sex was performed. Outcomes evaluated were in-hospital mortality and prolonged length of stay (LOS) ≥ 75^th^ percentile.

## Results

**Figure 1:**
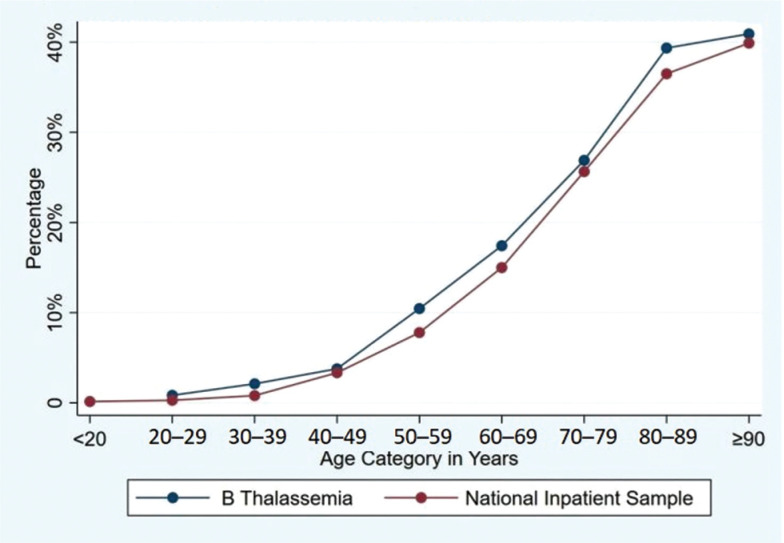
Prevalence of atrial fibrillation in β-thalassemia patients from the National Inpatient Sample.

A total of 17,150 admissions were included in the study period, of which 2100 (12.2%) admissions had AF. Admissions with AF were older (mean age, 72.1 vs. 47.3 years; *P* < .001); more likely to be White (66% vs. 41%); and more likely to have CHF, hypertension, valvular heart disease, and/or renal disease **(Table 1)**. BT admission was associated with a higher AF prevalence than non-BT admissions across all age groups **(Figure 1)**. AF was not associated with an increased risk of in-hospital mortality (adjusted odds ratio [aOR], 1.36; 95% confidence interval [CI], 0.67–2.78; *P* = .398) or an increased LOS (aOR, 1.00; 95% CI, 0.78–1.29; *P* = .997) in the general cohort. In the subgroup analysis according to sex, AF was associated with increased in-hospital mortality in women (aOR, 2.73; 95% CI, 1.09–6.8; *P* = .031) despite an unchanged LOS (aOR, 0.80; 95% CI, 0.56–1.14; *P* = .218). Increasing age and CHF were predictors of in-hospital mortality and prolonged LOS. Also, liver disease was a predictor of mortality, while diabetes mellitus (DM) was a predictor of prolonged LOS.

## Discussion

The prevalence of AF in the U.S. population is approximately 2% in people <65 years of age, while it is about 9% in people aged ≥65 years.^4^ Our study revealed that 12.2% of enrolled BT admissions had an associated diagnosis of AF, which is comparable to the findings of a study by Patsourakos et al. that estimated the prevalence of AF among BT patients to be around 10.64%.^5^ Another observational study showed an AF incidence of 14% in the BT cohort without cardiac dysfunction.^6^ Our study confirmed statistically significant differences in baseline characteristics between the AF and non-AF cohorts. BT admissions with AF were older (mean age, 72.1 vs. 47.3 years; *P* < .001) and more likely to be White (66% vs. 41%).

The major mechanism behind the higher prevalence of AF is myocardial iron overload from repetitive transfusions, leading to cardiomyopathy, atrial dilatation, and remodeling. A study showed that left atrium dilatation is more frequent in BT patients than in healthy controls and is considered an early feature of cardiac remodeling in BT patients that predisposes them to AF.^7^ Another study using echocardiography and magnetic resonance imaging showed that diastolic function remains abnormal even when myocardial iron concentrations are normal in BT patients.^8^

Kirk et al.’s study^9^ showed that patients with severe iron overload had a higher incidence of arrhythmic events at 1 year of follow-up (relative risk, 8.79; 95% CI, 4.03–19.2), and AF was the most frequent arrhythmia identified in these patients. This supports the association between iron deposition and the incidence of arrhythmias.^10^ Another study by Origa et al. showed the importance of adherence to therapy and determined that low compliance with chelation therapy was highly predictive of arrhythmias.^10^

Russo et al. studied the relationship between P-wave dispersion and the incidence of AF in BT patients and documented significantly higher values of P-wave duration (>111 ms) and P-wave dispersion (>35 ms) in BT patients compared to normal subjects. Furthermore, P-wave dispersion was also inversely related to iron deposition as assessed by magnetic resonance imaging.^11^

Our study revealed multiple poor outcome predictors in BT patients, including DM, increased age, and CHF, as was reported previously in the study by Pepe et al. examining the prediction of cardiac complications in thalassemia major patients.^12^ Many studies have shown a worse effect of comorbid DM and AF^13^; in particular, a certain study observed that DM is associated with worse AF symptoms, a lower quality of life, and an increased risk of death and hospitalizations.^14^ The same was true for CHF: the simultaneous presence of CHF and AF identifies individuals at a substantially increased risk of cardiovascular events and death.^15^

Hypertension is an independent risk factor for stroke, increasing the risk by about 3-fold. A systematic review by Dzeshka et al. showed that hypertension in AF patients doubled the risk of stroke (relative risk, 2.0; 95% CI, 1.6–2.5). Other large observational studies, such as the Swedish Atrial Fibrillation cohort analysis (n = 182,678), have confirmed the major independent role of hypertension in the prognosis of AF.^16^

The current study revealed that AF was independently associated with an increased mortality risk in female BT patients. AF leads to a higher risk for all-cause mortality, cardiovascular mortality, and cardiovascular events (including cerebrovascular events and CHF) in women than in men.^17,18^ Some studies have shown that female BT patients experience more cardiovascular events with risk factors.^19–21^ In the AF Follow-up in Rhythm Management (AFFIRM) trial, women had a higher risk of stroke than men even with a similar therapeutic anticoagulation range on warfarin.^18^ Female sex is also an independent risk factor for AF recurrence after either electrical cardioversion or ablation.^19,20^ The mechanism behind why women with AF experience risk factors differently from men is unclear.^22^ The authors believe that this is multifactorial. Women are less likely to be prescribed anticoagulation therapy and to receive anti-arrhythmic and ablation treatments for AF.^23–25^ Many sex-specific physiological differences may also contribute to the increase in adverse events in female patients. That is why more studies are needed to understand sex-related differences and reduce the disparities in female AF patients with BT.

### Limitations

Our study has certain limitations. First, this was a retrospective study that is subject to confounding bias despite rigorous statistical adjustment and utilization of different methods of analysis. Second, the administrative nature of the database and reliance on ICD-10-CM codes make it prone to coding bias, where certain diagnoses such as BT could be underreported. Third, information in the NIS database is limited to that gathered from index hospitalizations; thus, out-of-hospital outcomes could not be evaluated.

## Conclusion

We report several significant findings in the largest retrospective trial examining AF in BT patients. First, AF is more common in BT compared to the general population, irrespective of age. Second, AF was independently associated with increased mortality risk in women. Third, poor outcome predictors that the study identified were CHF, liver disease, DM, and increased age. Further research is needed to explain the impact of AF on mortality in female patients. Development of novel strategies is needed to improve the quality of care and outcome in this group of vulnerable patients.

## Figures and Tables

**Table 1:** Baseline Characteristics and Outcomes Stratified According to the Presence of AF

Variable	β-thalassemia (n = 17,150)		*P* Value
	AF Absence (n = 15,050)	AF Presence (n = 2100)	
Baseline characteristics			
Mean age in years (SD)	47.3 (11.4)	72.1 (8.4)	<.001
Female	10,675 (71%)	1190 (57%)	<.001
Race			<.001
White	6060 (41%)	1360 (66%)	
Black	4540 (31%)	260 (13%)	
Hispanic	1090 (7%)	85 (4%)	
Asian/Pacific Islander	1925 (13%)	240 (12%)	
Native American	110 (1%)	<11 (<1%)	
Other	930 (6%)	100 (5%)	
Teaching hospital	12,345 (82%)	1645 (78%)	.077
Primary insurance			<.001
Medicare	5050 (34%)	1605 (76%)	<.001
Medicaid	3710 (25%)	140 (7%)	
Private	5340 (35%)	315 (15%)	
Self-pay	500 (3%)	30 (1%)	
Other/no charge	445 (3%)	<11 (<1%)	
Chronic comorbidities			
Congestive heart failure	1855 (12%)	1130 (54%)	<.001
Valvular heart disease	775 (5%)	605 (29%)	<.001
Hypertension	5425 (36%)	1625 (77%)	<.001
Diabetes mellitus	3140 (21%)	730 (35%)	<.001
COPD	2955 (20%)	535 (25%)	.007
Renal disease	2280 (15%)	865 (41%)	<.001
Liver disease	925 (6%)	265 (13%)	<.001
Obesity	2250 (15%)	250 (12%)	.102
Alcohol abuse	320 (2%)	50 (2%)	.734
Outcomes			
In-hospital mortality	170 (1%)	95 (5%)	<.001
Median LOS in days (IQR)	3 (2–5)	4 (3–8)	<.001

*Abbreviations:* AF, atrial fibrillation; COPD, chronic obstructive pulmonary disease; IQR, interquartile range; LOS, length of stay; SD, standard deviation.
